# FT-IR versus EC-QCL spectroscopy for biopharmaceutical quality assessment with focus on insulin—total protein assay and secondary structure analysis using attenuated total reflection

**DOI:** 10.1007/s00216-020-02718-1

**Published:** 2020-06-01

**Authors:** Sven Delbeck, H. Michael Heise

**Affiliations:** Interdisciplinary Center for Life Sciences, South-Westphalia University of Applied Sciences, Frauenstuhlweg 31, 58644 Iserlohn, Germany

**Keywords:** Insulin stability, FT-IR-ATR spectroscopy, Quality control, Secondary structure analysis, Biopharmaceuticals, Insulin fibrils

## Abstract

For the quality control of biopharmaceutical products, which contain proteins as the most important active ingredients, shelf life may be limited due to inappropriate storage conditions or mechanical stress. For insulins as representatives of life-saving pharmaceuticals, analytical methods are needed, which are providing additional information than obtained by assays for total protein quantification. Despite sophisticated formulations, the chemical stability may be challenged by temperatures deviating from recommended conditions or shear rate exposure under storage, leading to misfolding, nucleation, and subsequent fibril formation, accompanied by a decrease in bioactivity. A reliable method for insulin quantification and determination of secondary structure changes has been developed by attenuated total reflection (ATR) Fourier-transform infrared spectroscopy of insulin formulations by a silver halide fiber-coupled diamond probe with subsequent dry-film preparation. A special emphasis has been placed on the protein amide I band evaluation, for which spectral band analysis provides unique information on secondary structure fractions for intact and misfolded insulins. Quantitative measurements are possible down to concentrations of less than 0.5 mg/ml, whereas the dry-film preparation delivers high signal-to-noise ratios due to the prior water evaporation, thus allowing a reliable determination of secondary structure information.

**Figure:**
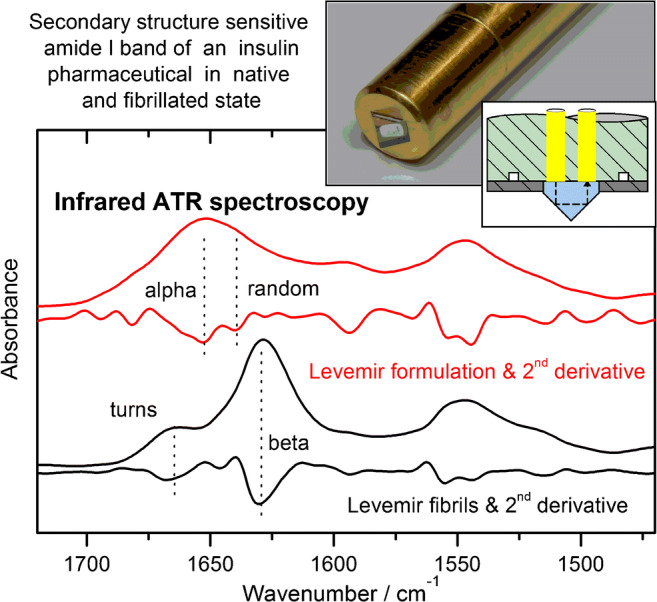
Graphical abstract

Graphical abstract

## Introduction

For the chemical analysis of commercial biopharmaceuticals for therapeutic usage and their bioactivity assessment, appropriate analytical methods are required, when concerned with proteins as the active ingredients and their excipients. Usually, formulated products include a collection of protein- or peptide-based therapeutics, which involve a wide range of products such as vaccines, blood components, e.g., interferons or growth factors, and recombinant therapeutic proteins. Therapeutic proteins can have enzymatic or regulatory and pharmacological activities. Human insulins and their analogs, which are representative members of the category of life-saving medications, are of great importance with a large market share within biopharmaceutical products. In particular, several aspects are to be taken into account, which are related to the identification and quantification of proteins and their stability assessment. In addition, the determination of protein higher-order structures, i.e., secondary and tertiary structures, is an important task during biopharmaceutical product development aiming in particular at lifecycle management. Biosimilar pharmaceutical drug development is another field of studies for higher-order analysis.

There are different challenges for the quantification and determination of the molecular stability in pharmaceuticals during production or later when obtained from pharmacies—especially after long-term storage under different environmental conditions that may be limiting their lifecycle. These adverse storage conditions can be associated with breaking the storage cold chain or during transport, encountered with shaking and elevated temperatures [1]. First deteriorating effects can be protein misfolding with drastic changes in the protein secondary structure, followed by protein aggregation and fibril forming leading to a reduction in the potency of the active pharmaceutical ingredient [2–4]. Aggregation can be followed by size-exclusion chromatography (SEC) [5] or by sodium dodecyl polyacrylamide gel electrophoresis (SDS-PAGE) [6]. Also, nuclear magnetic resonance (NMR) spectroscopy assays have been suggested for insulin aggregation studies [7, 8]. Another option is the application of dynamic light scattering (DLS) methods, which provide information on aggregation [9, 10].

Most analytical methods, i.e., high performance liquid chromatography (HPLC) with UV detection, HPLC coupled with mass spectrometry (MS) [11], NMR spectroscopy [8, 12], or a photometric Bradford assay [13], can be successfully utilized for the determination of total protein content, but these are unable to provide information on the secondary structure, which is essential for the assessment of biopharmaceutical protein (e.g., insulin) potency. Identification can be undertaken by mass spectrometry, mainly in combination with chromatography methods, but especially matrix-assisted laser desorption ionization time of flight mass spectrometry (MALDI TOF) is a candidate for the identification of insulins, available in whole formulations, by providing information on their molecular mass.

For protein secondary structure analysis, especially Fourier-transform infrared spectroscopy (FT-IR) and electronic circular dichroism (ECD) spectroscopy have been applied, as characteristic absorption bands at specific wavelengths can be exploited for this important key parameter in therapeutic proteins [14, 15]. The ECD measurement in the far-UV has been used, but is supposed to work only with low concentrations from 0.05 to 1 mg/ml and to be susceptible to excipients; thereby, a buffer exchange may be required. ECD is especially useful for α-helical protein analysis due to the intense signal that α-helix structures provide in the far-UV region. Other options exist also with vibrational circular dichroism (VCD) measurements. Even though such analyses, based on VCD, do not need to be limited to molecules containing chiral chromophores as in ECD, the experiments are more complex, including strong absorption of water in the amide I region as described below. The undesirably strong water absorption is usually avoided by using a shorter sample path length (∼ 6 μm) for standard transmission measurements.

For the analysis of protein secondary structures, particularly in the event of misfolding and subsequent possible fibrillation processes, infrared spectroscopy represents one of the most successful analytical tools (see, e.g., [16, 17]). Especially when analyzing the amide I band interval within the mid-IR range, FT-IR spectroscopy can resort to many publications and online databases, which are not available to our knowledge for Raman spectroscopy as an alternative method. Recently, external cavity quantum cascade lasers (EC-QCL) have been advocated for several different applications for protein analysis, because larger transmission cell path lengths could be utilized in comparison with conventional FT-IR spectroscopy. Even a commercially available instrument has been developed for biopharmaceutical quality assessment [18]. Further investigations on protein secondary structure analysis had been carried out by the Lendl group [19, 20]. Reasons for this are the larger intensities of the lasers in comparison with the thermal radiation sources, such as globars that are part of FT spectrometers. With intensive radiation sources as available with tunable EC-QCL, the spectral range around the protein amide I and II bands becomes accessible also for aqueous solutions, and several research groups have used such instrumental setup with transmission cells of 23 to 30 μm path length for protein secondary structure analysis. The availability of high spectral power density in the biological fingerprint region from 900 to 1700 cm^−1^ allows for mitigating the impact of the high absorption of water. A limitation of recent commercially available QCL instrumentation is however that the mid-infrared spectral scanning range is usually limited to ~ 250 cm^−1^ (which can differ), because of the limited tunability of the individual EC-QCL modules [21]. For FT spectrometers, when avoiding opaqueness connected with maximum band absorbances than larger three, transmission cells with a path length smaller than 10 μm have been advised for aqueous biofluid analysis such as the AquaSpec flow cell from Bruker Optics with a path length of ~ 7 μm. Besides quantitative measurements of proteins and other ingredients, which can be connected to the quality assessment of biopharmaceuticals, also quantification of secondary structure elements or changes can be achieved.

Other options are provided with attenuated total reflection (ATR) spectroscopy. ATR measurements of blood plasma have been performed earlier by using a μ-Circle cell with a 2.5-cm-long zinc selenide (ZnSe) rod and cone-shaped ends for radiation coupling by a special Axiom optics [22]. The accessory had been placed within the instrument sample chamber for routine transmission measurements. A transmission-equivalent path length at 1000 cm^−1^ was around 10 μm for allowing a restriction-free measurement within the mid-infrared region with water absorbance compensation. More flexible and allowing remote sensing are silver halide–coupled ATR elements based on diamond prisms or cones [23], a technology with an untapped potential, which will be presented here.

**Fig. 1:**
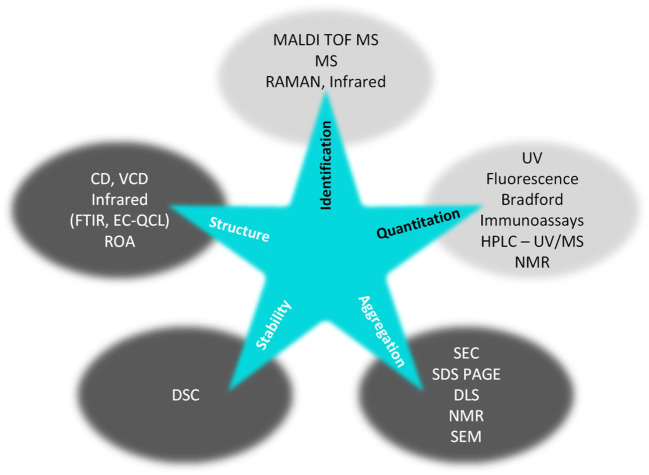
Overview of the different analytical approaches, used for the analysis of proteins. Besides the options on determining structural characteristics such as the secondary structure for monitoring the protein stability or aggregation processes, also a variety of possibilities for the quantitation and identification exist (SDS-PAGE, sodium dodecyl sulfate polyacrylamide gel electrophoresis; SEM, scanning electron microscopy; DSC, differential scanning calorimetry; ROA, Raman optical activity)

Reliable techniques for the determination of even smallest changes in the secondary structure of insulins and insulin analogs in their original formulations are available with spectroscopic methods. There is evidence that the secondary structure of the insulin peptide mainly influences its bioactivity and therefore it is assumable that structural changes lead to a decrease in its biological potency [24]. Early events in the globular insulin peptide’s misfolding during elevated stress and temperature events reveal changes in the peptide’s A and B chains, including the exposure of partially unfolded sites leading to intermediate monomeric states. These intermediates are enabling subsequent formation of beta sheet substructures and can be detected without changes concerning their amino acid sequence [3, 4]. Due to this possibility, for example, HPLC methods are able to detect both types of active and inactive insulins, but without discriminating between the two. Consequently, these methods are not useful for the determination of the physiologically active protein from the recorded chromatograms. In Fig. 1, an overview on different aspects of quality assessment and monitoring is given, highlighting the toolbox of analytical methods and their application strongholds.

## Experimental, chemicals, and methods

Different insulin formulations were purchased from pharmacies. In particular, results from Levemir® (insulin detemir, Novo Nordisk, Bagsvaerd, Denmark) and Humalog® (insulin lispro, Eli Lilly, Indianapolis, USA) are reported here. As reference, the United States Pharmacopoeia (USP) human insulin standard (Sigma-Aldrich, St. Louis, USA) was procured.

All insulin samples were stored in their original vials at recommended temperatures around 5 °C using a refrigerator [1]. The USP human insulin standard was dissolved in a standard phosphate buffer at pH = 7.4 according to Sörensen and stored in sealed plastic tubes, guaranteeing highest sterility. The same treatment was applied for the insulin detemir sample for forced fibril formation. Fibrillation was realized by adjusting a Levemir sample to pH 2.0 with 0.1 M hydrochloric acid and subsequent storage at 37 °C for 48 h in a climatic chamber. Scanning electron microscopic (SEM) images of the sample were recorded for manifesting fibril formation in the insulin formulation after the incubation time [25]. Pointing to changes in the secondary structure, shifts of the maximum amide I band position were observed in infrared spectra due to increasing beta sheet and beta turn fractions within the insulin samples with absorption bands around 1630 and 1665 cm^−1^, respectively.

**Fig. 2:**
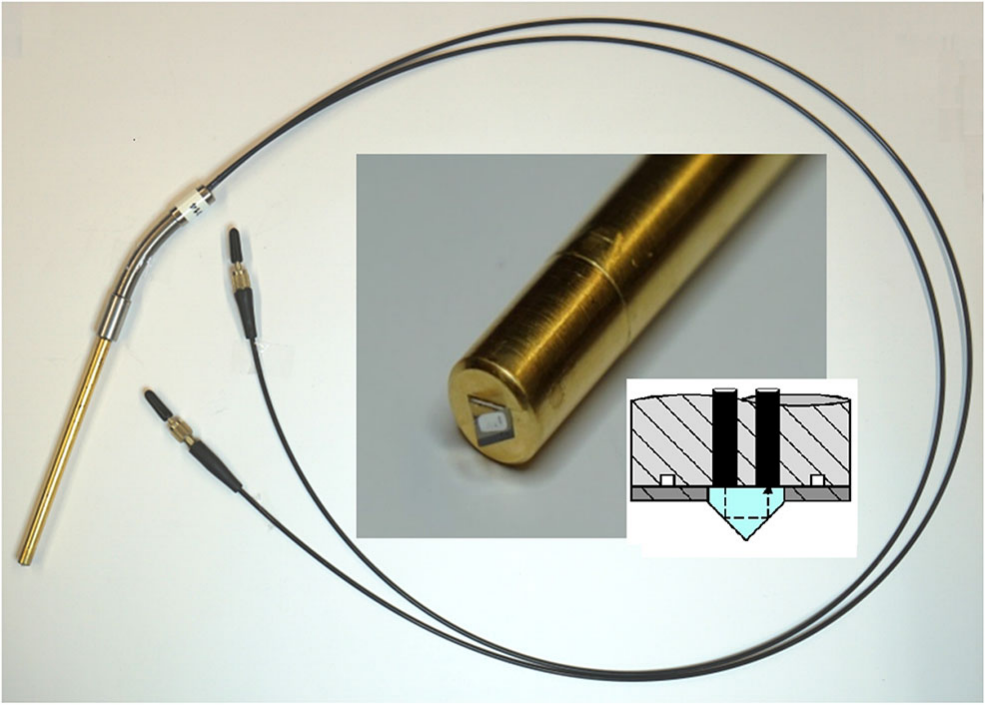
Photography of the fiber-optic probe with two silver halide waveguides encased in polyetheretherketone (PEEK) tubings for guaranteeing stability, flexibility, and protection from chemical and UV damage. The probe head in the inset shows the diamond ATR element allowing for two 45° reflections, as highlighted in the schematics

For a detailed analysis of the different commercial insulin formulations, all excipients were purchased at analytical grade (Sigma-Aldrich, St. Louis, USA) and prepared with known concentrations for insulin-free infrared background measurements (see Table 1). FT-IR-ATR measurements were carried out with a fiber-optic diamond probe (infrared fiber sensors, Aachen, Germany—see Fig. 2), allowing remote sample sensing and being attached to the side of the housing of a Vector 22 spectrometer (Bruker Optics, Ettlingen, Germany). The parallel infrared beam exiting the FT-IR spectrometer was coupled into an encased wave-guiding silver halide fiber by an off-axis parabolic mirror with a numerical aperture of 0.6. The fiber-coupled diamond ATR sensor element is furnishing two internal reflections within the diamond prism at 45° and can be immersed into the sample to be analyzed. Subsequent to the attenuation of the infrared radiation by the sample, a second fiber guides the beam to a pigtailed mercury cadmium telluride (MCT) photodetector, cooled by liquid nitrogen.

**Table 1:** Composition of two insulin analogs, including all excipients and the insulin concentration as stated in the product information from the European Medicines Agency, the package insert for information and direction for use and from Ref. [2]

Excipients	Levemir pH = 7.4	Humalog pH = 7.0–7.8
Insulin detemir	14.20 mg/dl	–
Insulin lispro	–	3.50 mg/dl
Glycerol	16.00 mg/dl	16.00 mg/dl
Phenol	1.80 mg/dl	trace
*m*-Cresol	2.06 mg/dl	3.15 mg/dl
Zn	654.00 mg/dl	197.00 mg/dl
Na_2_HPO_4_	1.12 mg/dl	2.36 mg/dl
NaCl, HCl, NaOH, water

Absorbance spectra of formulated insulin and reference excipients were recorded with a resolution of 4 cm^−1^, and Fourier transformation was applied using a Blackman-Harris 3-term apodization function with a zero fill as appropriate for a data point spacing of 1 cm^−1^. The total measurement time was approximately 2 min, resulting in 400 sample interferogram scans at ambient temperature. Different background spectra were used, while sample absorbance spectra were measured against the clean diamond ATR prism. Water absorbance compensated spectra were calculated with buffer solution or distilled water as spectral background. For the analysis of the insulin characteristic amide I bands between approximately 1700 and 1600 cm^−1^, second derivative spectra were calculated after a min–max normalization of the absorbance spectra to localize the band positions of the secondary structure single bands.

For the analysis of essential parameters of the infrared ATR measurements, reference solutions of 1000 mg/dl creatinine in distilled water were prepared and spectra recorded with the Bruker Vector FT-IR spectrometer using a 30 μm CaF_2_ transmission cell (inner volume 0.6 μl), as well as the fiber-optic diamond probe. Further comparison measurements between FT-IR and EC-QCL spectroscopy, both based on the ATR and transmission technique, were performed with aqueous glucose (100 mg/dl), bovine serum albumin (BSA, 15 mg/ml) and ethylenediaminetetraacetic acid (EDTA) solutions, human plasma, as well as associated protein-free dialysate samples (all chemicals purchased from Sigma-Aldrich, St. Louis, USA). The latter is useful for the analysis of aqueous constituents. The EC-QCL system was equipped with four gap-free tunable mid-infrared laser modules covering the spectral range between 1850 and 780 cm^−1^ (LaserTune, Block Engineering, Southborough, USA), a CaF_2_ transmission cell with a path length of either 50 or 100 μm and a thermoelectrically cooled MCT detector (for each module’s wavenumber range, see below).

**Fig. 3:**
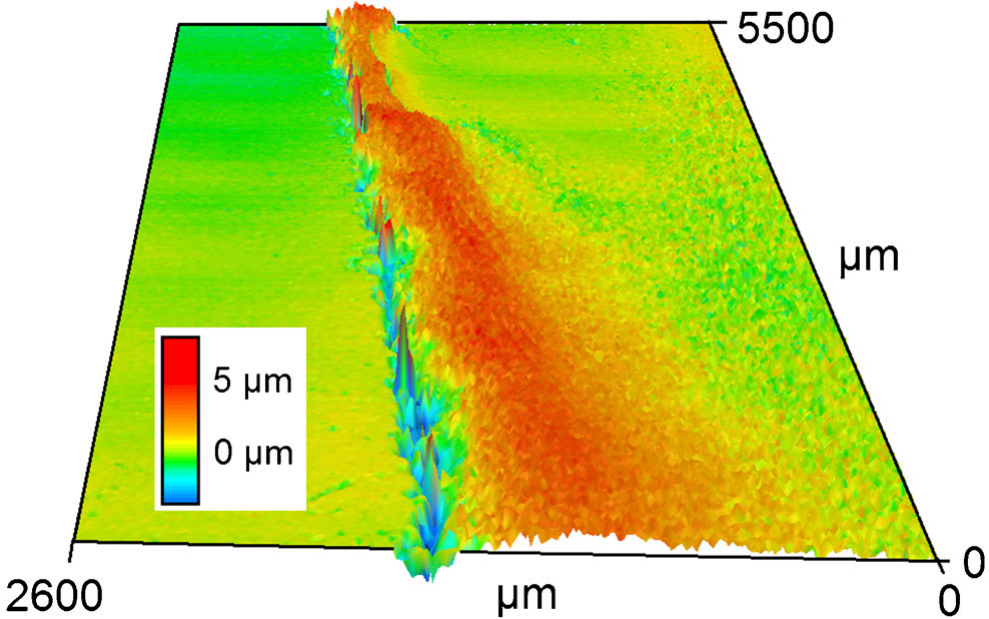
Image of the surface structure and thickness of a model insulin detemir dry-film, showing a cross-section on a microscopic slide, recorded with a laser scanning microscope (LSM). For the preparation, a 50 μl sample had been poured alongside the tape/slide interface in vertical direction and was then dried under ambient temperature in a laminar flow cabinet for 24 h, before the tape was removed (left side)

The stability and secondary structure of proteins in aqueous and dried states has often been discussed with different outcomes. For the determination of structural changes within the insulin molecule, the comparison of second derivative spectra of the same sample, either as dry-film or aqueous sample, is well suited. After submersion of the fiber-optic probe into the formulation, a high-quality spectrum of the insulin specimen can be recorded. Next, after withdrawal from the insulin-containing tube, the remaining film was air-dried. The drying process was monitored by the decreasing intensities of the water absorption band around 1650 cm^−1^ and the simultaneously increasing intensities of the amide I and II bands in the wavenumber range between 1680 and 1450 cm^−1^. For determining the quality of the insulin dry-film’s thickness and homogeneity, laser scanning microscopic images were recorded (VK-X 100, Keyence Corporation, Osaka, Japan) after the preparation of an insulin detemir dry-film on a microscopic glass slide. One part of it was covered by a tape strip, which was removed for revealing a cross-section at the interface insulin dry-film and glass slide (see Fig. 3). An average height of 3–5 μm was identified by using the Keyence analysis software, representing a solid base for further dry-film ATR measurements.

## Results

### Theoretical considerations for ATR measurements

The infrared ATR measurement technique represents a rather fast and easy approach for the analysis of organic compounds in all kinds of physical states. An extensive description of the ATR technique, including theoretical background, as well as practical applications, has been given by F.M. Mirabella [26]. In contrast to prevalent transmission spectroscopy, samples can directly be analyzed without any preparation. One of the technical requirements, and also the most important one, is that the sample has to be in direct contact with the optically denser ATR element; as for analysis, an evanescent wave penetrates the first few microns of the sample. For theoretical considerations, the depth-dependent attenuation of the evanescent wave intensity by the sample absorption, described by the electric field, needs to be investigated. For this, the sample’s complex refractive index is defined by ${\overset{\sim }{n}}_2={n}_2+i\ {k}_2$, where$i=\sqrt{-1}$ and *k*_2_ is the absorption index. Also, perpendicular- and parallel-polarized radiation needs to be considered for estimating the energy, absorbed by the sample. An approximation for the so-called sampling depth, representing the location where the electric field, originating from the element/sample interface, has decayed to a value of 1/e (~ 37%), can be given by the penetration depth (*d*_*p*_) equation:

1 $$ {d}_p\left(\upalpha, \lambda \right)=\lambda /\left(2\pi {n}_1\sqrt{\Big({\mathit{\sin}}^2\upalpha -{\left({\overset{\sim }{n}}_2/{n}_1\right)}^2}\right) $$

with

*λ*: radiation wavelength.
α: angle of incident radiation, larger than the critical angle for total reflection.
*n*_1_, $$ {\overset{\sim }{n}}_2 $$
refractive indices of both optical media with *n*_1_ > *n*_2._

The second important value for a quantitative characterization of ATR measurements is the “working depth,” where the square evanescent electric field has decayed to 0.25%, approximately given by a threefold of the aforementioned penetration depth. For the determination of the effective layer thickness for samples with only weak absorption, as used for comparing a transmission spectroscopic approach with that of an ATR measurement, Harrick has given the dependency of the transmission-equivalent layer thickness for the two kinds of polarized radiation [27].

2 $$ {d}_{\mathrm{perpendicular}}=\frac{\lambda \cdotp {n}_z\cdotp \cos \alpha }{\pi \cdotp \left(1-{n}_z^2\right)\sqrt{{\mathit{\sin}}^2\alpha -{n}_z^2}} $$

3 $$ {d}_{\mathrm{parallel}}=\frac{\lambda \cdotp {n}_z\cdotp \cos \alpha \cdotp \left(2{\sin}^2\alpha -{n}_z^2\right)}{\pi \cdotp \left(1-{n}_z^2\right)\left[\left(1+{n}_z^2\right){\mathit{\sin}}^2\alpha -{n}_z^2\right]\cdotp \sqrt{{\mathit{\sin}}^2\alpha -{n}_z^2}} $$

with $$ {n}_z={\overset{\sim }{n}}_2/{n}_1 $$

Under the assumption of same amounts of perpendicular- and parallel-polarized radiation, as found in unpolarized radiation, the effective layer thickness (*d*_*eff*_) can be calculated:

4 $$ {d}_{eff}=\left({d}_{\mathrm{perpendicular}}+{d}_{\mathrm{parallel}}\right)/2 $$

In case of an incident beam with a default reflection angle of α = 45°, the relation below is valid [26]:

5 $$ {d}_{\mathrm{perpendicular}}=\frac{1}{2}\ {d}_{\mathrm{parallel}} $$

### Experimental validation and practicality considerations

**Fig. 4:**
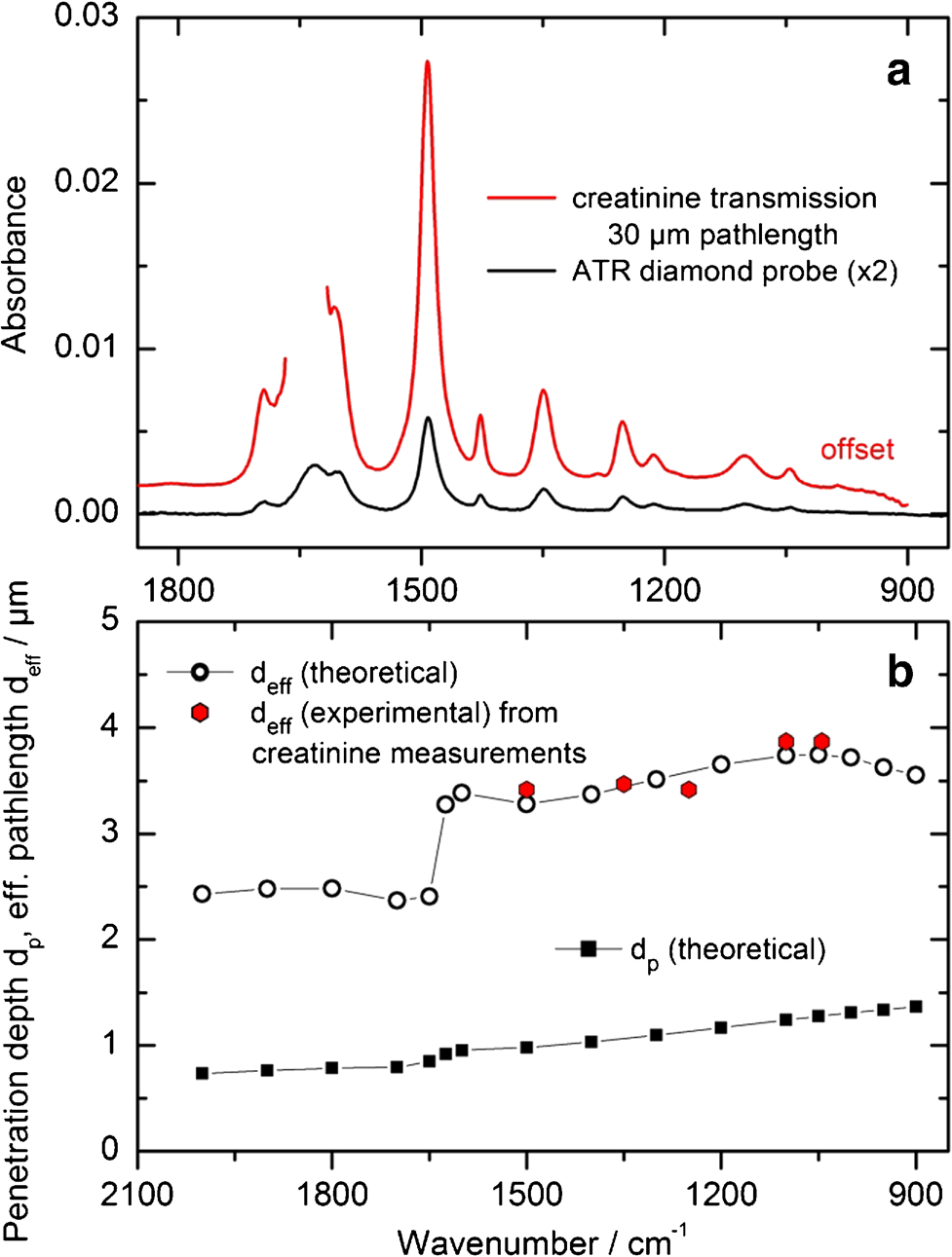
Comparison of spectra from 1% aqueous creatinine solutions as measured with a 30 μm transmission cell and an ATR diamond probe (**a**). Experimental and theoretical considerations concerning the penetration depth and effective path length for radiation in the mid-infrared wavenumber range between 2100 and 850 cm^−1^ into an aqueous diluted solution, using optical constants of water from Bertie et al. [30] (**b**)

Experimental results for the comparison of different infrared spectroscopic techniques were obtained from aqueous creatinine solutions, measured in transmission within a 30 μm cell and by using the ATR diamond probe with two internal reflections and a refractive index of *n*_diamond_ = 2.42 [28]. The spectral region between 1850 and 900 cm^−1^ is of special interest for analytical use (see Fig. 4a). Illustrated absorbance spectra with narrow characteristic vibrational bands from creatinine, offering a homogeneous absorption band distribution within the spectral fingerprint interval with appropriate maximum band absorbances also for 30 μm sample path length at the chosen concentration, can be used for an estimate of the effective layer thickness (*d*_*eff*_), as measured by the ATR technique and compared with a transmission experiment with suitable path length. For this, the maximum band intensities were ratioed. The gap in the red curve can be explained by the total absorption of radiation by water, measured as background spectra for all samples. In Fig. 4b, theoretical and experimental results are plotted for the calculated effective layer thickness, obtained from the diluted aqueous samples. Also, the theoretical penetration depth (*d*_*p*_) is shown (black squares). The saltus in the measured set of data points around 1650 cm^−1^, showing the effective layer thickness (open black circles), can be explained with the optical constants of water, as published by Bertie [29]. Experimental results show a very good agreement with the theoretical estimates for the presented data.

**Fig. 5:**
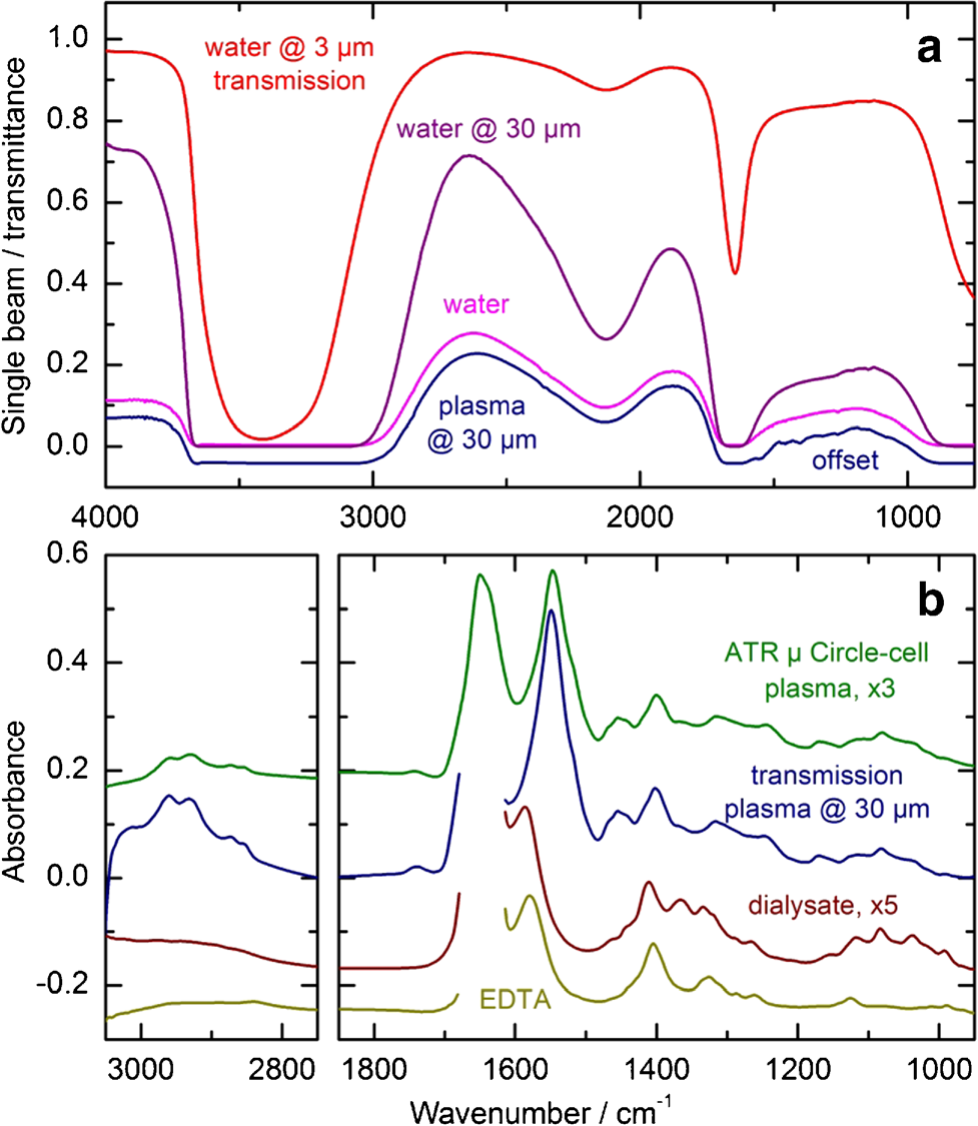
Transmittance (red and purple) and single beam (magenta and blue) spectra of distilled water and human plasma as measured in a transmission cell with a path length of either 3 or 30 μm (**a**). Absorbance spectra of a human plasma sample measured with an ATR μ-Circle cell (green) and in transmission (blue—30 μm path length). The dark red and yellow curves represent a dialyzed plasma sample and an ethylenediaminetetraacetic acid (EDTA) solution, measured in the 30 μm transmission cell (**b**)

For the analysis of biopharmaceuticals, especially the amide I region between 1700 and 1600 cm^−1^ needs to be accessible via infrared spectroscopy. In this region, the secondary structure-sensitive absorption bands of the protein are located, providing information on its biological activity when hormone cell receptor-specific bindings are taken into account, as in the case of insulin. The potency can be related to the uptake of glucose into the cells [24]. Accessibility of this spectral range can be achieved by reducing the water-specific absorption due to the OH bending vibrational band around 1650 cm^−1^ for managing absorbance compensation with acceptable signal-to-noise (S/N) ratios. When using a globar as radiation source, one option is the application of thin transmission cells (e.g., around 3 μm), leading to an approximate transmittance of 50% (see Fig. 5a), instead of facing total absorption when using a cell of 30 μm path length (purple curve). Single beam spectra of water and a human plasma sample, containing solved plasma proteins, are illustrated below and show the inaccessible spectral intervals between approximately 3700–3000 cm^−1^, 1700–1550 cm^−1^, and below 900 cm^−1^, leading also to the impossibility of analyzing the amide I bands.

Accordingly, absorbance spectra of human plasma samples (Fig. 5b), showing protein-related vibrational bands as measured by an ATR μ-Circle cell and by a 30 μm transmission cell, are able to demonstrate the advantages of the ATR measurement technique. It is obvious that the spectral region of the amide I band can be analyzed (green curve) in contrast to the same region in the spectrum when recorded in transmission (blue curve). Concerning the spectral range below 3000 cm^−1^, where symmetric and antisymmetric CH_2_ and CH_3_ stretching vibrations can be identified, spectral information is accessible with both techniques, as well as within the so-called fingerprint region. After dialysis of a human plasma sample for achieving a further reduction of the sample complexity by removing the plasma proteins, absorbance spectra were recorded, showing the absence of the amide II and III bands around 1540 and 1450 cm^−1^, respectively. Again, there is no information available from the spectral range around 1650 cm^−1^. The main component, as seen in the dialyzed blood spectrum (dark red curve), derives from ethylenediaminetetraacetic acid (EDTA), which was used to prevent coagulation in blood samples for analysis.

### FT-IR versus EC-QCL spectroscopy

**Fig. 6:**
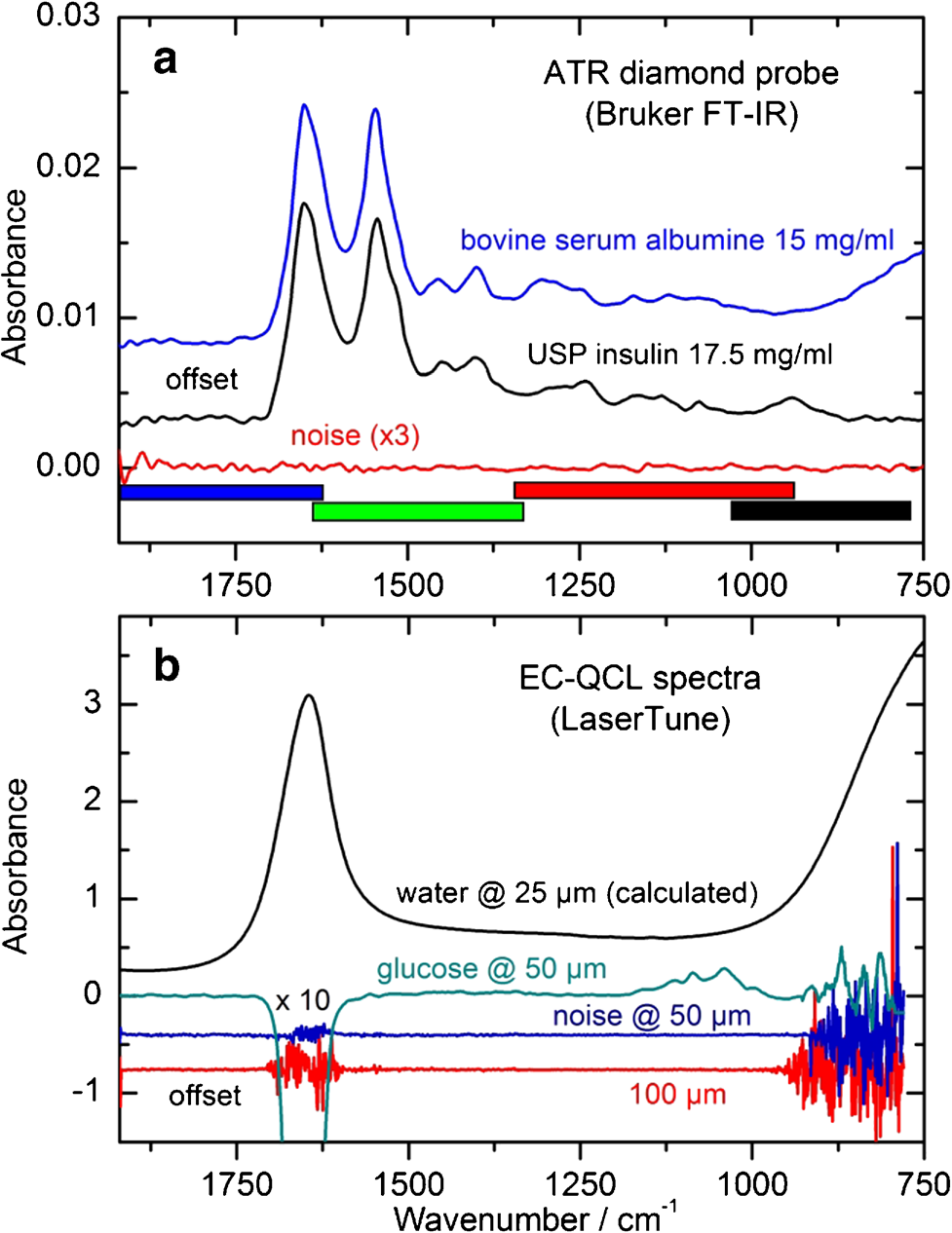
Comparison of two different infrared spectroscopic measurement techniques. In plot (**a**), aqueous solutions of bovine serum albumin and an USP human insulin sample were measured by ATR spectroscopy and reveal the potential for analyzing the whole protein spectrum between 1750 and 750 cm^−1^. The colored bars at the bottom represent the spectral ranges, covered by four combined tunable QCL modules, whose spectra are shown below (**b**). The black curve is a calculated water spectrum at a transmission path length of 25 μm. Plotted underneath is a glucose spectrum, measured in a 50 μm transmission cell by the EC-QCL spectrometer with an average power of 3 mW, as well as absorbance noise spectra for two water-filled cells with different path lengths

For another infrared measurement technique to be compared with the ATR FT-IR spectroscopic setup, the LaserTune EC-QCL spectrometer with different transmission cells was applied. At the bottom of Fig. 6a, the spectral ranges from the four tuneable laser modules are presented, showing the gap-free possibility of recording infrared spectra within the protein-representative region. Other devices with a single laser module are certainly available with adjusted scanning ranges for accessing the amide I and II band interval. For the same plot, two spectra from aqueous protein solutions (bovine serum albumin (BSA) and human insulin) with similar concentrations were measured with the diamond ATR FT-IR technique. Amide I, II, and III bands of both proteins are well detectable and can be analyzed regarding their secondary protein structure, with a maximum absorbance of around 0.015 absorbance units for the amide I band. An ATR spectrum of distilled water, measured against the same as background (red curve), shows a low rms noise value, as calculated to be 4.04 ∙ 10^−5^ within the spectral range of 1700–900 cm^−1^, leading to a limit of quantification (LOQ) as estimated to be around 0.5 mg/dl. In Fig. 6b, EC-QCL spectra are plotted, showing an aqueous 250 mg/dl glucose solution as measured in a 50 μm transmission cell, as well as the according noise spectrum together with another one from a 100 μm cell. Again, as already shown for the absorbance spectra when using a globar as radiation source, the spectral amide I range, revealing information on the secondary structure, is not accessible due to opaqueness from total absorption by water. Therefore, cell path lengths of around 25 μm have been suggested for protein characterization assays (for a corresponding water spectrum, see also the black curve, calculated from data by Bertie et al. [29]). To obtain this spectral information, either the transmission path length needs to be reduced to a few microns, as with FT-IR spectroscopy, or the increased power of the emitted QCL radiation has to be utilized, as demonstrated by Lendl et al. [19, 20].

### Quantification of insulin and excipients in formulations

**Fig. 7:**
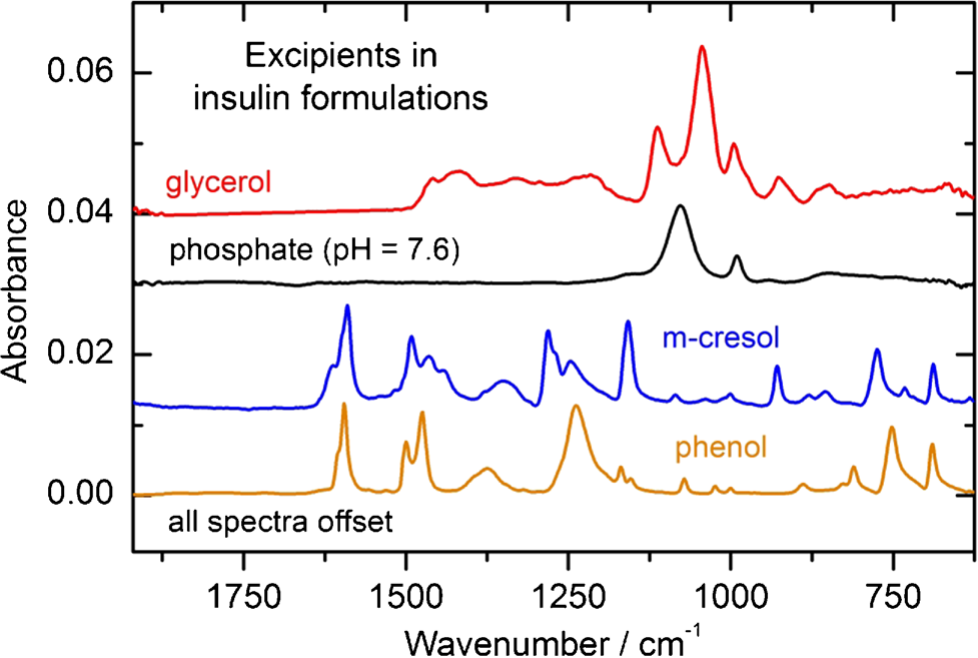
Spectral overview of the different aqueous excipients as used in commercial insulin formulations within the wavenumber range of protein-related vibrational bands. All spectra are offset for clarity and were measured against water as background with the fiber-optic ATR probe

Most human insulins and analogs in pharmaceutical formulations contain different stabilizing excipients, as listed in Table 1. Besides non-absorbing stabilizers and buffer chemicals in the mid-infrared spectral range like zinc ions, as used for insulin hexamer formation, others show contributions to the recorded spectra, due to vibrational bands also in the protein amide I region. Therefore, spectra of pure components, or spectra from excipients dissolved in distilled water, were recorded and are presented in Fig. 7. Especially aqueous solutions of m-cresol and phenol show distinct vibrational bands around 1590 cm^−1^, resulting in confounding bands between the amide I and amide II interval and need to be considered when analyzing protein secondary structure using insulin formulations. The pH-dependent phosphate spectrum (black curve) has two main absorbance bands around 900 and 1100 cm^−1^ and therefore would not influence the structure-relevant protein bands. The same seems to be the case for glycerol, but here, hydrogen bonds can link between the OH groups of water and glycerol, resulting in an additional band close to the water absorption band above 1650 cm^−1^, as localized in the second derivative spectrum of insulin Levemir as discussed later.

**Fig. 8:**
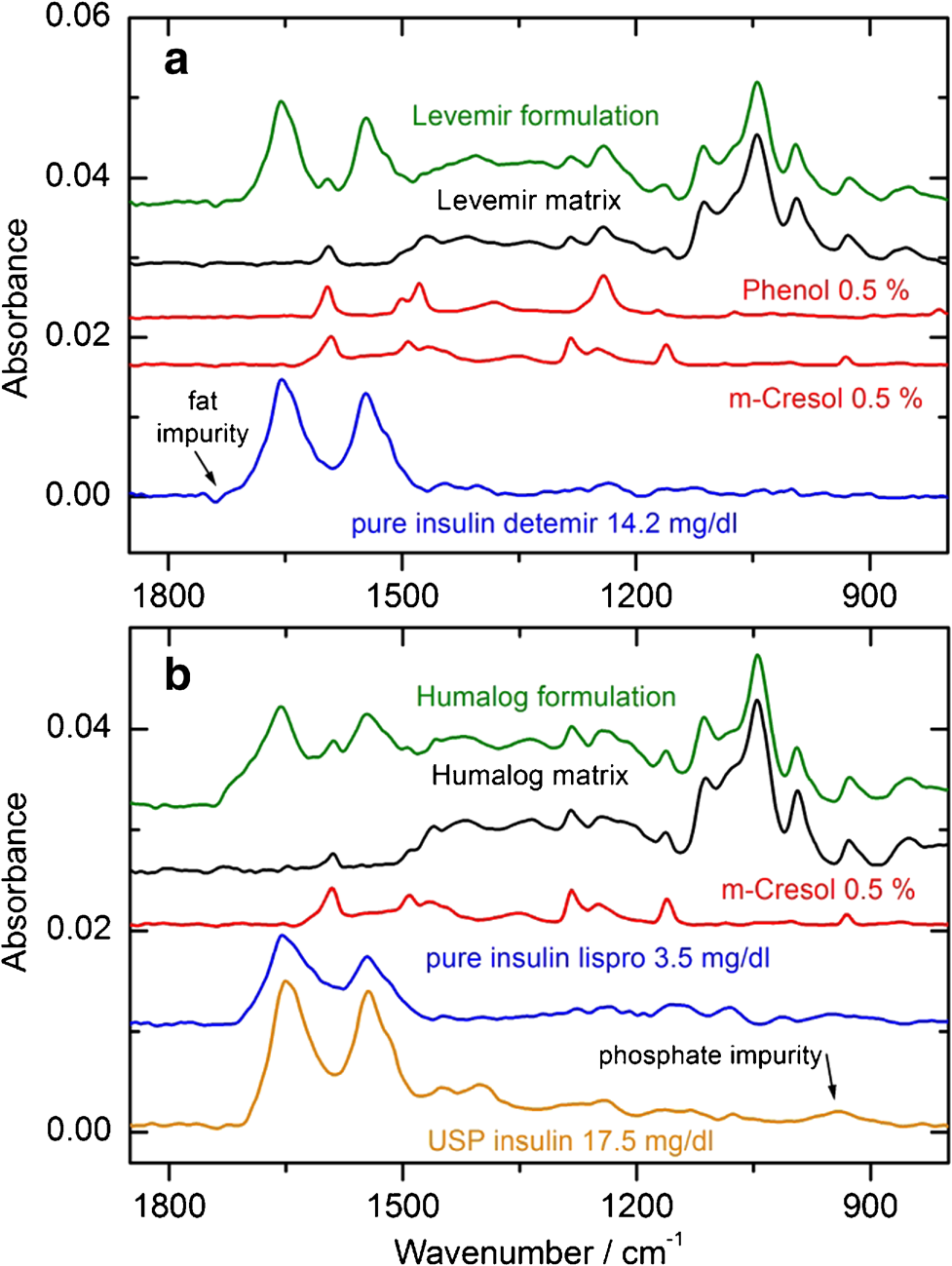
Plots (**a**) and (**b**) show a detailed spectral analysis of two commercially available insulins, such as insulin detemir (Levemir) and insulin lispro (Humalog), respectively. For the matrix composition, the excipients with appropriate concentrations were taken from the package insert (see Table 1)

Next, the analysis of two commercial insulins, i.e., insulin detemir (Fig. 8a) and insulin lispro (Fig. 8b) is presented. The infrared ATR measurements have been carried out with the fiber-optic diamond probe and include original formulations, the corresponding excipient matrices as prepared from known concentrations in distilled water, spectra of interfering aromatic stabilizers, as well as pure analog insulin spectra after scaled subtraction. Quantitative information on the original Levemir formulation can be obtained from spectral signatures of phenol and m-cresol, glycerol, and absorbing phosphate as matrix components. The vibrational bands of the biopharmaceutical matrix sample have a perfect match with those of the original formulations. This offers an easy approach for quality monitoring of pure insulin detemir (blue curve). The same procedure was successful for the Humalog insulin sample, which is compared with the reference USP insulin sample from a solution in phosphate buffer, illustrating matching protein-specific bands.

This situation allows for multivariate data analysis for the determination of all constituents, including the precise quantification of the insulin concentration in original formulations. One option is the use of a classical least squares (CLS) method, where high-quality reference spectra with known concentrations, as seen in Fig. 8, are to be applied. Due to hydrogen bonding effects between water and glycerol, CLS methods need additional attentiveness within the spectral region around 1000 cm^−1^, which will be discussed in the following section. Another option is certainly the application of partial least squares (PLS) analysis, which requires formulated samples with varying concentrations of all constituents for calibration [22, 30, 31]. An advantage, compared with the CLS method, is the inclusion of compounds with pH-dependent vibrational bands, e.g., as experienced for the inorganic phosphate spectra. For a spectral library of different insulins with special focus on the amide I and II spectral range, principal component analysis (PCA) can be used for the identification of different human and analog insulins, based on comparing scores from the insulin-specific signatures [32].

### Quality analysis of pure and formulated insulins

**Fig. 9:**
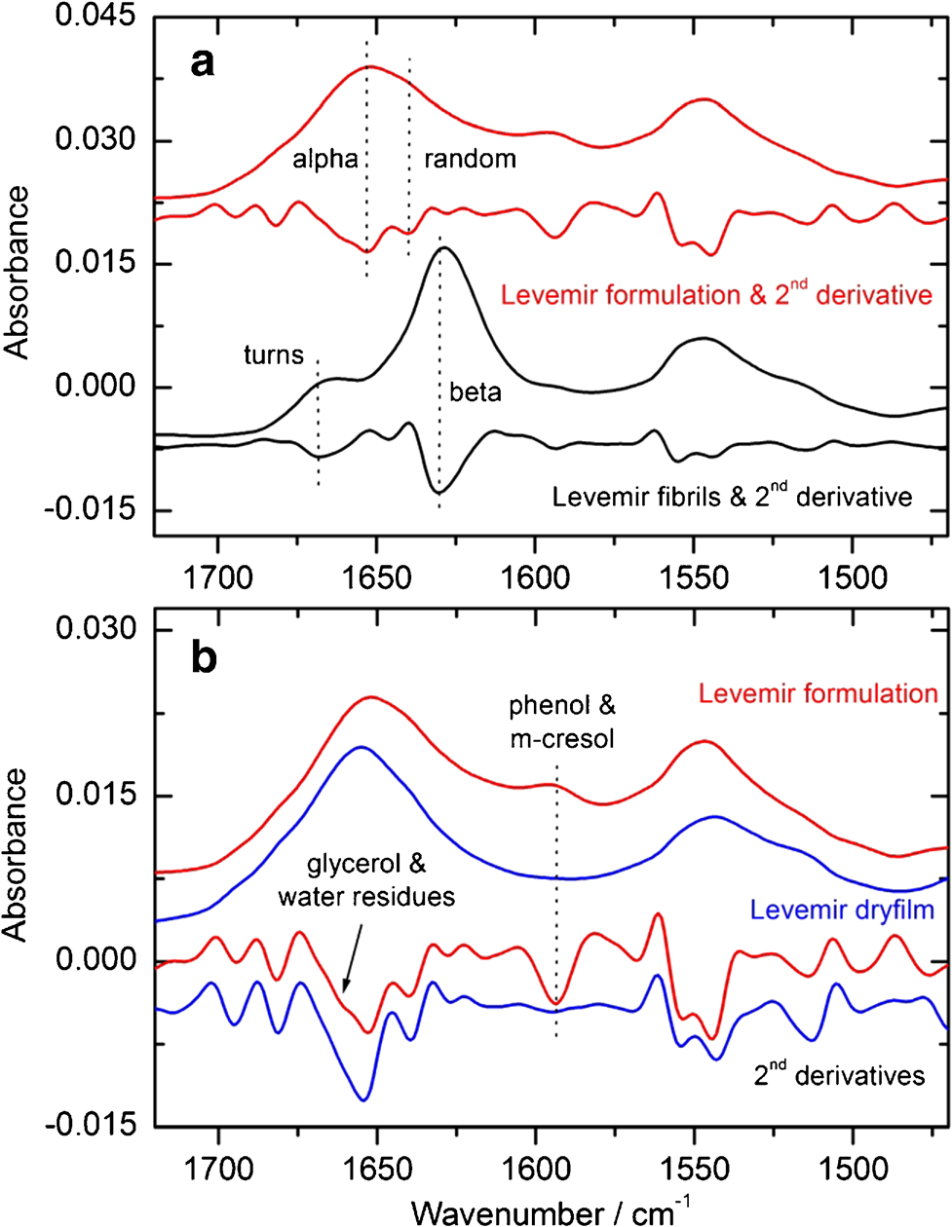
(**a**) Spectra of a formulated insulin detemir (Levemir), freshly taken from the vial (red), and after fibrillation (black), as well as the corresponding second derivative spectra. (**b**) Comparison of absorbance and second derivative spectra from a formulated and dried insulin detemir sample (scaled for clarity), showing the same protein secondary structures

For a presentation of the potential of the suggested analytical methods for protein structure analysis, which is an essential aspect for quality monitoring of insulins, examples with measurement-specific features are illustrated in this section. The upper plot in Fig. 9 shows two spectra, recorded with the fiber-optic probe, where the red curves derive from a freshly drawn sample of insulin detemir (Levemir) and the black curves represent the sample with fibrils formed from the same insulin specimen. For both cases, absorbance spectra are accompanied by their second derivatives, as calculated with a nine point smoothing Savitzky-Golay algorithm. Based on the locations of curve minima, individual absorbance bands, representing insulin secondary substructures, can be determined and ratios for misfolded fractions approximated through band deconvolution and integration [25]. For the fresh insulin sample, mostly alpha helical structures can be detected, whereas for the insulin fibrils, a distinct shift of the main band from 1655 to 1630 cm^−1^ is obvious, representing beta sheet structures, as well as another band around 1670 cm^−1^, which can be assigned to beta turn secondary structures [33]. For the evaluation of an add-on measurement technique with insulin dry-films, which is providing significantly increased signal-to-noise ratios, spectra of the same sample were recorded and compared (see Fig. 9b). The upper absorbance spectra show great conformity, which can further be manifested by the second derivative features, highlighting the same secondary structure information from both spectra. Differences are obvious around 1660 cm^−1^, which can be explained by the already mentioned hydrogen-bonded complexes, resulting from glycerol and water mixtures, as obvious in the aqueous sample (red second derivative curve). Another distinct band, visible in the absorbance and the second derivative spectrum of the insulin formulation, reveals information on the presence of phenol and m-cresol between the amide I and II bands. These characteristic signatures disappear while preparing dry-films of the samples, due to evaporation of the mentioned excipients.

Both measurement techniques provide high-quality spectra of insulins in their formulations and can therefore be used for a reliable and sensitive characterization of the secondary structure, as derived from amide I vibrational bands. A sensitive assessment of changes within the protein is possible by the comparison of second derivative spectra, pointing to even smallest variations within the hormone’s secondary structure. Based on the identified subband wavenumber locations, contributions from individual substructures, e.g., alpha helix, beta sheet, random coil, and beta turns, can be calculated by band deconvolution and individual subband integration. In addition, a shift of amide I band maxima can be used as a first evidence for secondary structure changes, as it moves from the alpha helical subband, as main constituent around 1650 cm^−1^, to the beta sheet band with its absorption maximum around 1630 cm^−1^ (see also Fig. 9a) [25].

**Fig. 10:**
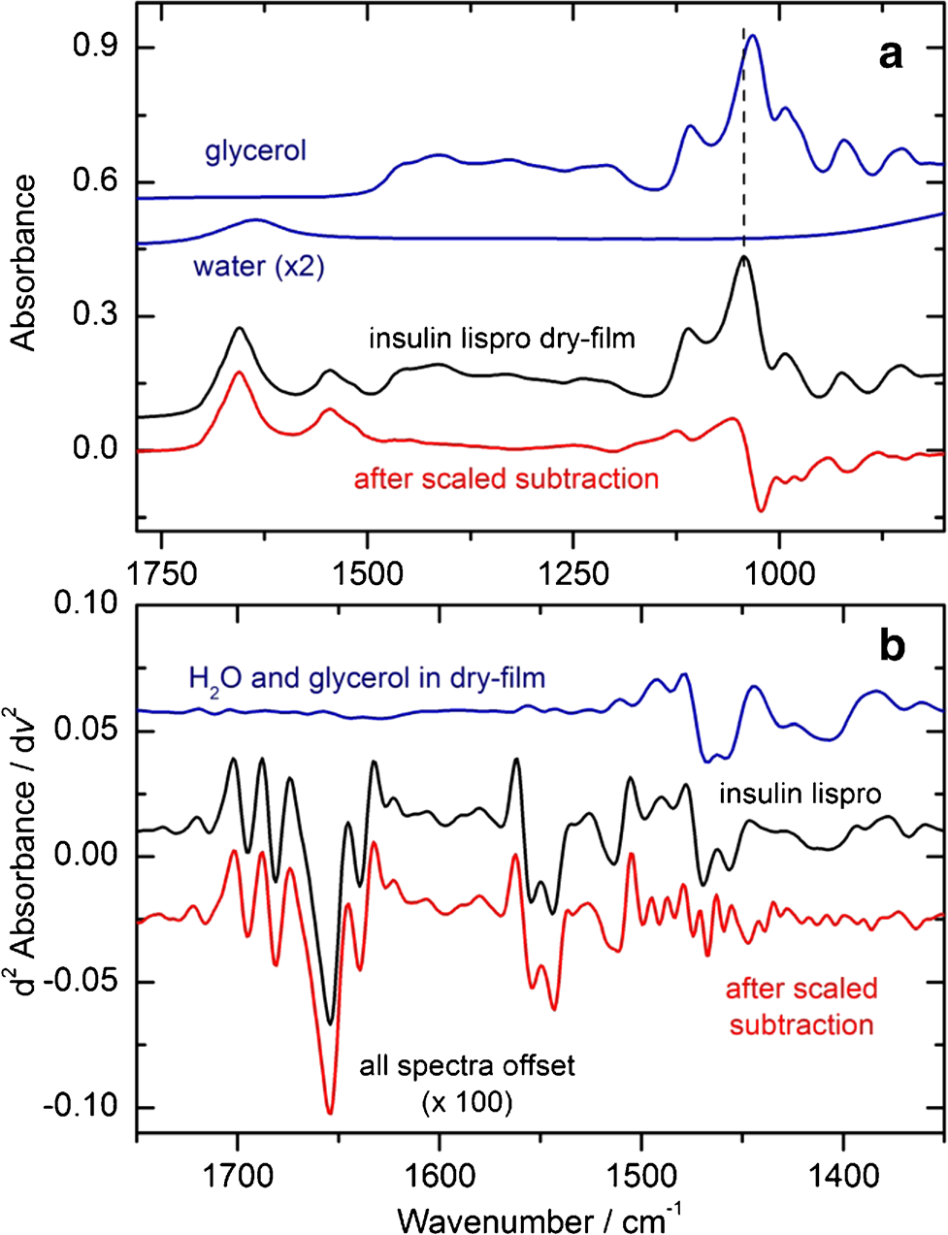
(**a**) Spectra of pure glycerol, water, and an insulin lispro dry-film sample, as well as the insulin sample after scaled subtraction, measured with the ATR fiber probe. (**b**) Second derivative spectra of an assembled water and glycerol spectrum, as characteristic for these excipients in formulated insulin dry-films, as well as from the insulin lispro sample before and after scaled subtraction of the abovementioned excipients

In Fig. 10, the high quality of insulin dry-films, as prepared from 1 μl formulation samples, is demonstrated. As mentioned above, the included aromatic excipients evaporate, which means that only residues from glycerol/water signatures will be present in the measured dry-films. Figure 10a shows a good example for the difficulties in the spectral analysis when it comes to the subtraction of aqueous glycerol spectral features. Due to hydrogen bond–related changes between pure glycerol and aqueous glycerol, spectral bands show a shift within the wavenumber range around 1000 cm^−1^ (blue and black curves), indicated by the dashed line. The red spectrum shows an insulin lispro sample after a scaled subtraction, illustrating the obvious glycerol band shifts also after a previous scaled subtraction of phosphate bands in the same spectral range. For determining the influence of water- and glycerol-related absorption within the amide I area of dry-film samples, second derivative spectra were calculated from an insulin lispro sample (black curve), as well as from a spectrum obtained from known amounts of aqueous glycerol and distilled water (blue curve; see Fig. 10b). After a scaled subtraction from the insulin lispro sample, there is evidence that spectral effects from glycerol and water are negligible when it comes to the quantitative analysis of the amide I band from insulin dry-film spectra (see red curve).

## Discussion

The quality monitoring of proteins, as used in biopharmaceuticals, requires highest standards for a reliable assessment of total protein concentrations, its stability, as well as for the characterization of the secondary structure. The USP general chapter <1049> about “Quality of biotechnological products: Stability testing of biotechnological/biological products” will be upgraded with the USP chapter <1049.1> “Design of stability studies for biotechnology product development and lifecycle management,” focusing on methodological strategies for the product stability testing also under stress conditions [34–36]. As an example for commercially available life-sustaining biopharmaceuticals, insulin and its analogs, as formulated with different chemical stabilizers, show all of the aforementioned analytical challenges.

Infrared spectroscopy is known for its possibilities with regard to extensive protein analytics and is therefore the method of choice, when using the appropriate measurement technique. For the simultaneous determination of total protein content in aqueous solutions and the analysis of the secondary structure, the protein-specific vibrational interval between approximately 1700 and 1500 cm^−1^ needs to be accessed. This requirement is a challenge to routine absorbance measurements with the application of transmission cells of defined path lengths, as used in conventional FT-IR spectrometers, equipped with a thermal radiation source. Here, limits by either the intensity of radiation or by a poor signal-to-noise ratio when using thin sample layers can be seen. As a possibility for solving this problem, the use of EC-QCLs, providing higher laser power, has been suggested but which can show deficits in reproducing radiation and intensities at default wavelengths, where careful wavelength calibration is needed [21, 37].

This is certainly a great advantage of FT-IR spectrometers with a stable wavenumber scale over the whole accessible mid-infrared spectral range. Disadvantages from transmission spectroscopy can also be neglected when using the ATR technique. In particular, the diamond fiber-optic probe offers an easy and flexible approach with one sample dip and dry-film formation from low volume samples for analyzing biopharmaceuticals in their original formulation with concentrations down to 0.5 mg/ml. The simultaneous quantification of formulated insulin content together with structure analysis can thus be performed in consecutive steps. In the special case of commercial insulin formulations, concentrations are usually above 3 mg/ml, so that multivariate quantification by IR-ATR spectroscopy, used as a standard analytical method, can be reliably achieved as demonstrated for even more complex sample compositions that were observed in blood plasma [22]. Recently, an infrared spectroscopic assay for total protein based on dry-film preparations on a rough surface polytetrafluoroethylene (PTFE) film for transmission measurements has been presented, for which amide I band integration has been employed for quantification and compared with, e.g., a Bradford photometric assay [38]. However, liquid sample measurements provide a much higher accuracy and reproducibility of the analytical results.

When unstable proteins begin to form aggregates or even fibrils with a possible inhomogenization process within the sample, this will lead to decreasing results concerning total protein content as experienced with HPLC methods and photometric assays. Regarding international regulations for the quality control of insulins, concentrations between 95 and 105% are acceptable for insulin formulations, so that unstable pharmaceutical products, as detected by FT-IR-ATR spectroscopy, will be rejected before entering the market. Besides the quantification, which is only one aspect of our suggested method, reliable and reproducible secondary structure analysis of different insulins can be carried out by calculating second derivative spectra after taking care of the infrared-active excipients within tested commercial insulin formulations and by deconvolution of the absorbance bands by band fitting, requiring high signal-to-noise ratios.

## Conclusion

Our suggested infrared ATR method can successfully be applied for total protein quantification, as in most insulin formulations, this hormone is the only active protein ingredient. Besides those, special consideration is needed for, e.g., NPH (neutral protamine Hagedorn) insulins, containing the protamine peptide as modifier. The dry-film measurement technique is offering a superb tool for the detection of secondary structure alterations, as observed from eventual breaks in the cold chain until reaching the customer or patient. Regarding economic requirements for analytical quality laboratories in the industry and for research purposes, conventional FT-IR spectrometers, compared with latest EC-QCL devices, impress with unbeatable price-performance ratios and come with matching software, offering the analytical tools used for, e.g., protein analytics. Besides that, also infrared analytical equipment, such as the fiber-optic diamond probes, is available and can directly be attached to the FT-IR spectrometer.
